# Relapse of follicular lymphoma arising from a non‐t(14;18) clone

**DOI:** 10.1002/jha2.28

**Published:** 2020-06-03

**Authors:** Yasuyuki Otsuka, Momoko Nishikori, Masakazu Fujimoto, Kensuke Nakao, Masakatsu Hishizawa, Hironori Haga, Akifumi Takaori‐Kondo

**Affiliations:** ^1^ Department of Hematology and Oncology Graduate School of Medicine Kyoto University Kyoto Japan; ^2^ Department of Diagnostic Pathology Kyoto University Hospital Kyoto Japan

**Keywords:** clonality, cytogenetics, lymphomas

## Abstract

Intraclonal diversity is commonly observed in patients with follicular lymphoma (FL), whereas tumor cells at the onset and relapse usually share early genetic events such as VDJ rearrangement of the immunoglobulin genes and t(14;18) translocation. We report a case of FL with relapse with FL that was clonally different from the tumor cells at onset. A 59‐year‐old male presented with paraaortic lymph node swelling and thickening of the right renal pelvic and ureteral wall was histologically diagnosed as FL, grade 1. Karyotypic analysis revealed t(14;18)(q32;q21) with +12 and +der(18)t(14;18). Ten years after the initial diagnosis, he suddenly developed systemic lymphadenopathy as a second relapse, and histological examination led to the diagnosis of FL grade 3B with diffuse large B‐cell lymphoma. Surprisingly, karyotypic analysis demonstrated the presence of +12 and 3q27 abnormality, which was proved to be a BCL6 translocation by fluorescence in situ hybridization, but the absence of t(14;18)(q32;q21). We compared VDJ rearrangement of the FL cells at onset and relapse and found that they were completely independent of each other. These tumor cells sharetrisomy 12 as a common genetic abnormality, and it is speculated that trisomy 12 may have occurred earlier than BCL2 and BCL6 translocations. These results suggest that there can even be cases of “relapse” of FL with an independent origin of the primary tumor cells. Our observation highlights the importance of re‐biopsy of relapsed FL, especially when it occurs after a long remission with different clinical presentation from that at the onset.

Follicular lymphoma (FL) is the most common subtype of indolent lymphoma and is characterized by the presence of a t(14;18)(q32;q21) chromosomal translocation. However, about 10‐30% of patients with FL do not have this translocation, and some of these t(14;18)‐negative cases alternatively carry *BCL6* translocation involving 3q27 [1]. FL with *BCL6* translocation has several distinct biological features, and it is associated with higher histological grade, atypical protein expression, and increased risk of histologic transformation [2, 3].

Although the survival of patients with FL has been improving for several decades, histologic transformation develops with an incidence of 2‐3% per year and is associated with poor outcomes. Recent comprehensive genetic studies have revealed that transformed FL not only evolves directly from the initial FL cells but often arises by divergent evolution from a common precursor through the acquisition of distinct genetic abnormalities [4, 5, 6]. Even in these cases, primary FL and transformed FL cells usually share VDJ rearrangement and early genetic abnormalities such as t(14;18) translocation. We report here a case of FL with relapse with FL that was clonally different from the primary tumor cells.

A 59‐year‐old male with obesity, hypertension, type 2 diabetes mellitus, hyperuricemia, and hyperlipidemia was found to have paraaortic lymph node swelling and thickening of the right renal pelvic and ureteral wall by [^18^F]‐fluorodeoxy‐d‐glucose (FDG) positron emission tomography‐computed tomography (PET‐CT) scanning at a medical checkup (Figure 1A). Cytological examination of the urine and ureteroscopic biopsy of the ureteral wall failed to show abnormal findings. A retroperitoneal laparoscopic biopsy of the paraaortic lymph node demonstrated nodal proliferation of small centrocyte‐like cells, which were positive for CD20 (clone L27, Roche, Basel, Switzerland), CD10 (clone 56C6, Leica, Deerfield, Illinois, USA), Bcl‐2 (clone 124, Dako, Tokyo, Japan), and Bcl‐6 (clone GI191E/A8, Roche), and diagnosis of FL, grade 1 was made (Figure 1B). Flow cytometric analysis revealed that the lymphoma cells were positive for CD19, CD20, CD10, IgM, IgD, Igκ, and HLA‐DR, and karyotypic analysis revealed t(14;18)(q32;q21) with +12 and +der(18)t(14;18) (Figure 1C). Fluorescence in situ hybridization (FISH) analysis confirmed the presence of *BCL2‐IGH* translocation, with most cells carrying three fusion signals (Figure 1D), which were compatible with the result of karyotyping. Because the patient demonstrated right hydronephrosis, he was treated with four cycles of R‐CVP (rituximab, cyclophosphamide, vincristine, and prednisone) followed by four cycles of rituximab monotherapy, and obtained complete remission (CR). However, his disease gradually relapsed in his right renal pelvis and ureter. Four years after the initial diagnosis, the patient received six cycles of BR (bendamustine and rituximab), which led him to durable CR. Ten years after the initial diagnosis, he suddenly developed progressive bilateral cervical lymph node swelling with elevation of serum lactate dehydrogenase to 522 IU/L (normal range, 124‐226 IU/L). PET‐CT scan revealed systemic lymphadenopathy with strong FDG uptake especially in the cervical, axillar, and mediastinal lymph nodes (Figure 2A). Histological examination of the biopsied cervical lymph node demonstrated follicular and diffuse (50%) proliferation of mostly centroblast‐like cells, and CD21‐positive follicular dendritic cell networks were only partially identified. Lymphoma cells were positive for CD20 and Bcl‐2, but weakly positive for Bcl‐6 and negative for CD10, and Ki‐67 positivity was 60% (Figure 2B). According to these findings, the diagnosis of FL grade 3B with diffuse large B‐cell lymphoma (DLBCL) was made. Flow cytometric analysis revealed that the tumor cells were positive for CD19, CD20, IgA, Igλ, and HLA‐DR. Surprisingly, karyotypic analysis demonstrated the presence of 3q27 abnormality and +12 but the absence of t(14;18)(q32;q21) (Figure 2C). FISH analysis confirmed the *BCL6* split signal, which indicated the presence of *BCL6* translocation, but a *BCL2‐IGH* fusion signal was not detected (Figure 2D), suggesting that the tumor cells were derived from a clonally different cell origin from the primary FL cells. With written informed consent of the patient, we compared VDJ rearrangement of the FL cells at onset and relapse using seminested polymerase chain reaction (PCR) and Sanger sequencing [7], and we found that they were completely independent of each other (Figures 1E and 2E). A summary of the characteristics of lymphoma at onset and relapse is shown in Table 1. The patient was treated with six cycles of R‐CHOP (rituximab, cyclophosphamide, doxorubicin, vincristine, and prednisone) and obtained a third CR.

**FIGURE 1:**
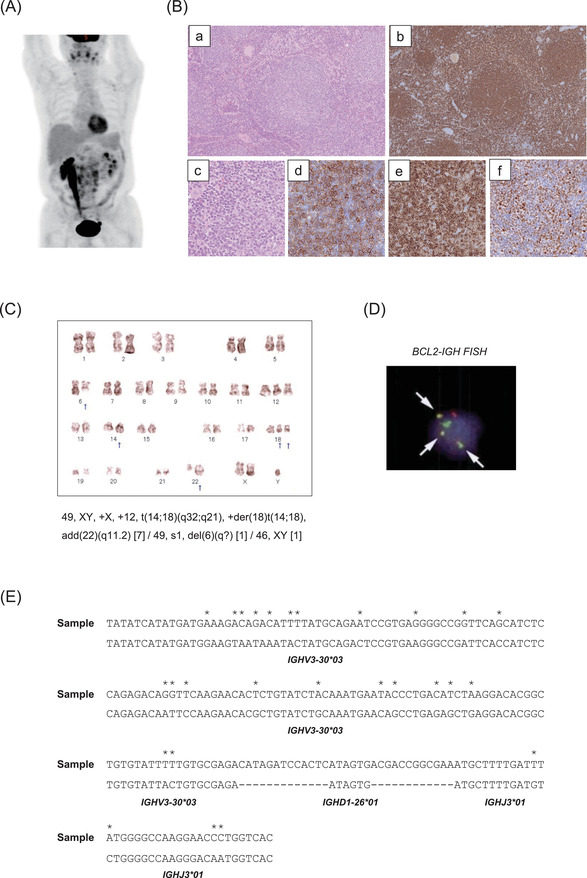
Clinical, pathological, and genetic features of lymphoma at onset. (A) PET‐CT scan of the patient. (B) Histological findings of the paraaortic lymph node, which was diagnosed as follicular lymphoma, grade 1. (a) Hematoxylin and eosin, (b) CD20, (c) Hematoxylin and eosin, (d) CD10, (e) Bcl‐2, (f) Bcl‐6. (Original magnification: a‐b, × 100; e‐f, × 400). (C) Karyotyping of lymphoma cells (SRL Inc. Tokyo, Japan). (D) Fluorescence in situ hybridization for *BCL2‐IGH* fusion signals (SRL Inc.). *IGH* is labeled with a green probe and *BCL2* is labeled with a red probe. Fusion signals are shown as yellow signals, which are indicated by arrows. (E) VDJ sequence of lymphoma cells at onset. DNA was extracted from formalin‐fixed, paraffin‐embedded lymphoma sample using NucleoSpin DNA FFPE XS (Macherey‐Nagel, Duren, Germany). The tumor‐specific VDJ sequence of the *IGH* gene was determined using seminested PCR and Sanger sequencing. Somatic hypermutations are indicated by asterisks

**FIGURE 2:**
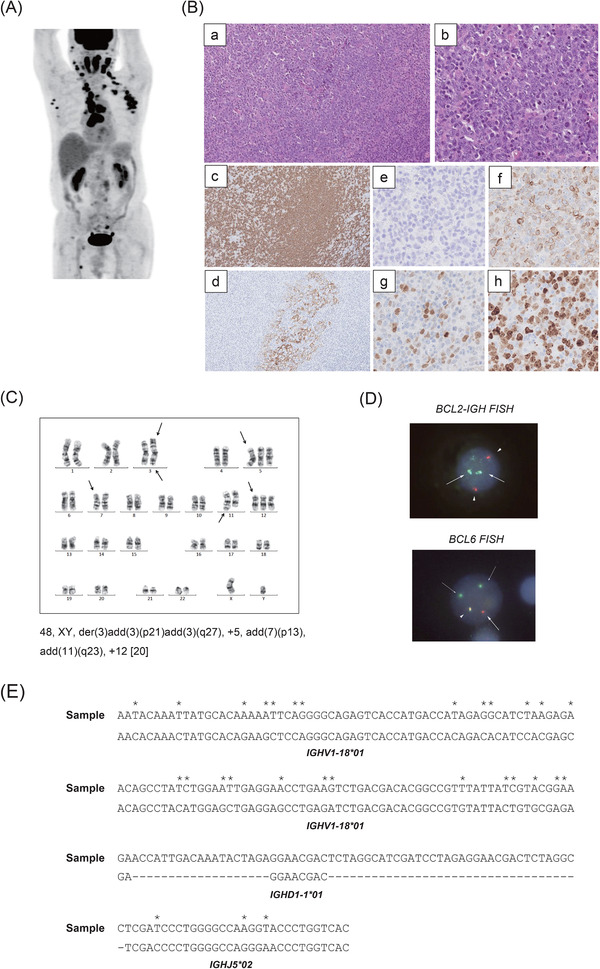
Clinical, pathological, and genetic features of lymphoma at relapse. (A) PET‐CT scan of the patient. (B) Histological findings of the cervical lymph node, which was diagnosed as follicular lymphoma, grade 3B. (a, b) Hematoxylin and eosin, (c) CD20, (d) CD21, (e) CD10, (f) Bcl‐2, (g) Bcl‐6, and (h) Ki‐67. (Original magnification: a, c d, × 100; b, e‐f, × 400). (C) Karyotyping of the lymphoma cells (LSI Medience Corporation, Tokyo, Japan). (D) Fluorescence in situ hybridization (FISH) for *BCL2‐IGH* fusion signals (upper panel) and *BCL6* split signals (lower panel) (LSI Medience Corporation). *IGH* and *BCL2* are labeled as in Figure 1D, but fusion signals were not detected. *BCL6* FISH demonstrated the split of red 5’*BCL6* probes (mostly two copies) and green 3’*BCL6* probes (mostly one copy) indicated by arrows, with one yellow unsplit signal indicated by an arrowhead. (E) VDJ sequence of lymphoma cells at relapse. DNA was extracted from fresh frozen lymphoma sample using a QIAprep Spin Miniprep Kit (Qiagen, Hilden, Germany), and tumor‐specific VDJ sequence of the *IGH* gene was determined as in Figure 1E

**TABLE 1:** A summary of the characteristics of lymphoma at onset and relapse

	Lymphoma at onset	Lymphoma at relapse
Patient's age	59	69
Tumor distribution	Paraaortic LNs, pelvic and ureteral wall	Cervical, supraclavicular, axillar, mediastinal LNs
Serum LDH	Normal (214 IU/L)	Elevated (522 IU/L)
Biopsied sample	Paraaortic LN	Cervical LN
Histology	FL grade 1	FL grade 3B with DLBCL
Flow cytometry	CD19, CD20, CD10, IgM, IgD, Igκ positive	CD19, CD20, IgA, Igλ positive; CD10 negative
G‐banded karyotype	49, XY, +X, +12, t(14;18)(q32;q21), +der(18)t(14;18), add(22)(q11.2) [7]/49, s1, del(6)(q?) [1]/46, XY [1]	48, XY, der(3)add(3)(p21)add(3)(q27), +5, add(7)(p13), add(11)(q23), +12 [20]
FISH analysis	*BCL2‐IGH* fusion signal positive	*BCL2‐IGH* fusion signal negative, *BCL6* split signal positive
*IGH* usage	*IGHV3‐30−IGHD1‐26−IGHJ3*	*IGHV1‐18−IGHD1‐1−IGHJ5*

DLBCL, diffuse large B‐cell lymphoma; FISH, fluorescence in situ hybridization; FL, follicular lymphoma; *IGH*, immunoglobulin heavy chain; LDH, lactate dehydrogenase; LN, lymph node.

Detailed comparison of FL at onset and relapse of this patient provides several important implications. The patient developed two clonally independent FL metachronously, and there may be some underlying predisposition to FL in this patient. There are several case reports describing the appearance of a different lymphoma clone in the clinical course of a patient with immunodeficiency [8, 9], or the development of a new lymphoid tumor as a secondary malignancy, but the patient was immunocompetent and did not have a history of intensive chemotherapies. The patient had several possible risk factors reported for FL such as sedentary lifestyle, obesity, preference for a diet rich in meat and alcohol [10]. In contrast, the patient did not have any history of exposure to chemicals, such as pesticides, industrial solvents, or hair dyes, which were reported as possible environmental risk factors for FL.

FL cells at both onset and relapse demonstrated trisomy 12 as a common genetic abnormality, and trisomy 12 may have occurred earlier than *BCL2* and *BCL6* translocations. Trisomy 12 is a recurrent genetic abnormality in chronic lymphocytic leukemia (CLL) and is also broadly detected in B‐cell malignancies [11, 12], whereas it was supposed as a secondary genetic change in some cases. There are two case reports in which trisomy 12 was shown to be shared in two clonally different B‐cell malignancies [13, 14]. It can be speculated that trisomy 12‐positive cells may serve as a common tumor precursor in different B‐cell malignancies, although further analysis is necessary. We were not able to detect a trisomy 12‐positive cell population by FISH in the patient's peripheral blood after chemotherapy that would support the hypothesis of the presence of a common precursor for lymphomas.

A wide range of clinical presentations and consequences of FL in patients has generally been attributed to the intraclonal heterogeneity of FL cells. However, we have shown that there can even be cases of “relapse” of FL with an independent origin of the primary tumor cells. This manuscript aimed to invite a discussion on the possible predisposition for the development of FL before t(14;18) generation. In any case, our observation highlights the importance of rebiopsy of relapsed FL, especially when it occurs after a long remission with different clinical presentation from that at the onset.

## CONFLICT OF INTEREST

The authors declare no potential conflict of interest in this work.

## References

[1] Bosga‐Bouwer AG , van Imhoff GW , Boonstra R , van der Veen A , Haralambieva E , van den Berg A , et al. Follicular lymphoma grade 3B includes 3 cytogenetically defined subgroups with primary t(14;18), 3q27, or other translocations: t(14;18) and 3q27 are mutually exclusive. Blood 2003;101(3):1149‐54. 12529293 10.1182/blood.V101.3.1149

[2] Akasaka T , Lossos IS , Levy R . BCL6 gene translocation in follicular lymphoma: a harbinger of eventual transformation to diffuse aggressive lymphoma. Blood 2003;102(4):1443‐8. 12738680 10.1182/blood-2002-08-2482

[3] Karube K , Guo Y , Suzumiya J , Sugita Y , Nomura Y , Yamamoto K , et al. CD10‐MUM1+ follicular lymphoma lacks BCL2 gene translocation and shows characteristic biologic and clinical features. Blood 2007;109(7):3076‐9. 17138820 10.1182/blood-2006-09-045989

[4] Pasqualucci L , Khiabanian H , Fangazio M , Vasishtha M , Messina M , Holmes AB , et al. Genetics of follicular lymphoma transformation. Cell Rep. 2014;6(1):130‐40. 24388756 10.1016/j.celrep.2013.12.027 PMC4100800

[5] Okosun J , Bodor C , Wang J , Araf S , Yang CY , Pan C , et al. Integrated genomic analysis identifies recurrent mutations and evolution patterns driving the initiation and progression of follicular lymphoma. Nat Genet. 2014;46(2):176‐81. 24362818 10.1038/ng.2856 PMC3907271

[6] Carlotti E , Wrench D , Matthews J , Iqbal S , Davies A , Norton A , et al. Transformation of follicular lymphoma to diffuse large B‐cell lymphoma may occur by divergent evolution from a common progenitor cell or by direct evolution from the follicular lymphoma clone. Blood 2009;113(15):3553‐7. 19202129 10.1182/blood-2008-08-174839

[7] Ramasamy I , Brisco M , Morley A . Improved PCR method for detecting monoclonal immunoglobulin heavy chain rearrangement in B cell neoplasms. J Clin Pathol. 1992;45(9):770‐5. 1401205 10.1136/jcp.45.9.770 PMC495101

[8] Barriga F , Whang‐Peng J , Lee E , Morrow C , Jaffe E , Cossman J , et al. Development of a second clonally discrete Burkitt's lymphoma in a human immunodeficiency virus‐positive homosexual patient. Blood 1988;72(2):792‐5. 3401598

[9] Kanno H , Ohsawa M , Iuchi K , Nakatsuka S , Yamamoto S , Nishioka M , et al. Appearance of a different clone of Epstein‐Barr virus genome in recurrent tumor of pyothorax‐associated lymphoma (PAL) and a mini‐review of PAL. Leukemia 1998;12(8):1288‐94. 9697886 10.1038/sj.leu.2401087

[10] Ambinder AJ , Shenoy PJ , Malik N , Maggioncalda A , Nastoupil LJ , Flowers CR . Exploring risk factors for follicular lymphoma. Adv Hematol. 2012;2012:626035. 23028387 10.1155/2012/626035 PMC3458409

[11] Johansson B , Mertens F , Mitelman F . Cytogenetic evolution patterns in non‐Hodgkin's lymphoma. Blood 1995;86(10):3905‐14. 7579360

[12] Schraders M , de Jong D , Kluin P , Groenen P , van Krieken H . Lack of Bcl‐2 expression in follicular lymphoma may be caused by mutations in the BCL2 gene or by absence of the t(14;18) translocation. J Pathol. 2005;205(3):329‐35. 15682435 10.1002/path.1689

[13] Nakamine H , Masih AS , Sanger WG , Wickert RS , Mitchell DW , Armitage JO , et al. Richter's syndrome with different immunoglobulin light chain types. Molecular and cytogenetic features indicate a common clonal origin. Am J Clin Pathol. 1992;97(5):656‐63. 1575210 10.1093/ajcp/97.5.656

[14] Peterson JF , Shah R , Kobrinski D . Two distinct BCL2 rearrangements, each observed in 2 independent subclones, evolving from a founder clone with trisomy 12 in a unique case of chronic lymphocytic leukemia/small lymphocytic lymphoma. Acta Haematol. 2017;137(4):237‐9. 28514780 10.1159/000474926

